# Wolf Is Back: A Novel Sensitive Sentinel Rejoins the *Trichinella* Cycle in the Western Alps

**DOI:** 10.3390/vetsci10030206

**Published:** 2023-03-09

**Authors:** Carlos Martínez-Carrasco, Barbara Moroni, Anna García-Garrigós, Serena Robetto, Emanuele Carella, Simona Zoppi, Paolo Tizzani, Moisés Gonzálvez, Riccardo Orusa, Luca Rossi

**Affiliations:** 1 Departamento de Sanidad Animal, Facultad de Veterinaria, Campus de Excelencia Internacional Regional “Campus Mare Nostrum”, Universidad de Murcia, 30100 Murcia, Spain; 2 Istituto Zooprofilattico Sperimentale di Piemonte, Liguria e Valle d’Aosta, Via Bologna 148, 10154 Torino, Italy; 3 Istituto Zooprofilattico Sperimentale di Piemonte, Liguria e Valle d’Aosta, Centro di Referenza Nazionale Malattie Animali Selvatici (CERMAS), Località Amerique 7G, 11020 Quart, Italy; 4 Department of Veterinary Sciences, University of Turin, Largo Paolo Braccini 2, 10095 Grugliasco, Italy; 5 Departamento de Sanidad Animal, Grupo de Investigación en Sanidad Animal y Zoonosis (GISAZ), Universidad de Córdoba, 14014 Córdoba, Spain

**Keywords:** Alps, Italy, *Trichinella britovi*, trichinellosis, wolf, carnivore, zoonosis, foodborne parasite

## Abstract

**Simple Summary:**

Trichinellosis is a foodborne disease caused by the nematode *Trichinella.* Human trichinellosis represents a public health hazard with a great socioeconomic impact in food safety, and it occurs by consumption of raw or undercooked infected meat. As apex predators, wolves may represent important actors in maintaining this parasite, although to date, there is a lack of information on *Trichinella* prevalence in the Western Alps where wolves have recently made return. The main aim of this study was to investigate the occurrence of *Trichinella* infection in grey wolves and discuss the epidemiological role played by this apex predator in the early phases of their return. During the period 2017–2022, we analyzed 130 muscle samples from individual wolves found dead in the Western Alps. *Trichinella* larvae were found in 15 wolves. Results suggest that, after recolonization of Northern Italy, the wolf has rejoined the *Trichinella* cycle and already plays a sensitive sentinel role for this zoonotic pathogen. The possible role played as new maintenance host is discussed and knowledge gaps are highlighted.

**Abstract:**

*Trichinella* is a foodborne parasite whose wildlife reservoirs are represented by carnivores and omnivores with predatory and scavenger behavior. The aim of the present study was to investigate the occurrence of *Trichinella* infection in grey wolves (*Canis lupus*) that recolonized the Western Alps from the end of the past century, and discuss the epidemiological role played by this apex predator in the early phases of its return. During the period 2017–2022, diaphragm samples were obtained from 130 individuals collected in the frame of a wolf mortality survey. *Trichinella* larvae were found in 15 wolves (11.53%) with a parasite intensity of 11.74 larvae per gram. *Trichinella britovi* was the only species identified. This is the first prevalence survey of *Trichinella* in wolves recolonizing the Alps. Results suggest that, in this particular biotope, the wolf has rejoined the *Trichinella* cycle and has the potential to play an increasingly important role as maintenance host. Arguments in favor and against this perspective are discussed and knowledge gaps highlighted. The calculated *Trichinella* larval biomass in the estimated wolf population roaming in Northwest Italy will serve as baseline value to explore possible shifts in the relative importance of wolves as *Trichinella* reservoir within the regional carnivore community. Finally, wolves re-colonizing the Alps already appear as sensitive sentinels to monitor the risk of *Trichinella* zoonotic transmission by infected wild boar meat.

## 1. Introduction

*Trichinella* spp. is a zoonotic nematode complex that may result in outbreaks affecting meat-eaters at the global scale. Recent *Trichinella* taxonomy comprises ten species and three additional genotypes, encompassing an ample host range of more than 100 animal species, that includes a majority of mammals but also birds and reptiles [1]. All taxa are characterized by a direct life cycle regionally supported by one or more maintenance hosts, usually wild carnivores and/or omnivores with a predatory and scavenger behavior, including wild and domestic swine amongst others [2]. As known, pig represents a common source of human trichinellosis (hereafter “trichinellosis”), whereas a “bridge-host” (a host that humans will consume more frequently than a carnivore) is needed to justify an appreciable zoonotic risk from wildlife sources [3,4]. In Europe, this risk is most often associated to the consumption of raw or undercooked wild boar meat and meat products [5].

The grey wolf (*Canis lupus*) is an apex carnivore of remarkable conservation interest, notoriously extirpated from vast areas of its Palaearctic range between the 18th to early 20th century. In the last decades, however, the scattered wolf populations that survived in Europe are again increasing in number and spreading in several countries, thanks to a mix of favorable contingencies (e.g., the abundance of wild ungulate prey) strengthened by effective conservation policies implemented since the second half of the 20th century [6]. Regarding the Alps, no wolf was present since the 1920–1930’s, when even the westernmost part of these mountains (eventually corresponding to our study area) was cleared of the last survivors. However, since the last decade of the 20th century, wolves migrating from peninsular Italy started recolonizing the Western Alps on both sides of the French-Italian border, and a new reproductive population successfully re-established here over the last 20 years [7]. According to recent estimates, a minimum of 946 wolves (CI 95%: 822–1099) and 104 reproductive packs are now deemed present in Northern Italy, over 41,600 km^2^, and further strengthening of this population in the Central and Eastern Alps is ongoing [8]. There is wide consensus that the growth of ungulate prey populations, in abundance and diversity, has been a major driver of wolf re-establishment in the Alps. However, if it is undisputable that wild ruminants and wild boar represent the main wolf prey throughout Europe [9,10], carnivores also appear in several diet studies, including red fox (*Vulpes vulpes*), dog (*Canis familiaris*) and golden jackal (*Canis aureus*) [11]. In addition, cannibalistic behavior (e.g., total or partial scavenging on conspecifics, or the consumption of a killed wolf on the intraguild conflict scene) has been reported, though often not adequately documented [12]. All these characteristics make the wolf an appropriate sentinel host for monitoring the circulation of several *Trichinella* genotypes/species [13,14], and a candidate to a substantial eco-epidemiological role as *Trichinella* maintainance host.

By definition, mammalian carnivores are *Trichinella* sentinels. They provide first line essential information on the regional presence/absence of these pathogens and the risk that bridge-hosts may become infected [15,16]. This, in turn, may inform the effort to prevent outbreaks of human trichinellosis of sylvatic origin and optimize the use of resources [17]. Studies have shown that reporting a trichinellosis outbreak by *T. britovi* in any country in Europe does not imply, in that particular country, a diffuse risk that a bridge-host, e.g., hunted wild boars, may harbor *Trichinella* larvae. In Italy, for instance, surveillance on thousands of red foxes, the recognized principal maintenance host of *T. britovi*, suggests that such risk is concentrated in mountainous zones and is null to irrelevant in lowland and hill areas countrywide [18]. In addition, long-term surveillance in wildlife in the Italian Alps has highlighted a progressive, still largely unexplained decline of *T. britovi* prevalence, and a regional shift from a hyperendemic to low endemic status of this infection in foxes over 50 years [19,20].

In this frame, as carnivores at the top of the food-chain, wolves may offer complementary information to traditional mesocarnivore-based surveys [21,22]. Moreover, in the mentioned context of rising wolf abundance at the continental scale, the growing number of wolf carcasses (e.g., those deriving from road collisions) available for diagnostic purposes may represent an opportunity to investigate the circulation of *Trichinella* spp. at the European wildlife level and, consequently, to have an indicator species to assess epidemiological aspects of this zoonosis. In Europe, *Trichinella* infection in wolves has been the object of several surveys, most of them carried out in the first two decades of the current century (see Table 1 for details on sample size, origin, typified agent/s, prevalence and intensity of muscle larvae).

Interestingly, infection was found in all investigated wolf populations and countries, with prevalence ranging from 3.8 to 100%, and parasite intensity from 0.009 to 250 larvae per gram (LPG). Four *Trichinella* species were involved (*T. britovi*, *T. spiralis*, *T. nativa* and *T. pseudospiralis*), with an expected northern distribution of *T. nativa* (in Scandinavia, Baltic countries and Russia) and a wider distribution of *T. britovi*, embracing from southern to Central and Eastern European countries up to southern Finland. *T. spiralis* in wolves has been reported less frequently and limited to Finland [35], Central Balkans [14], Croatia [21] and Germany [36], though never in Italy. Regarding *T. pseudospiralis*, there is only one case described in European wolves, specifically in Central Italy [37].

Taking advantage of a remarkable number of necropsies that we carried out to monitor the natural and human-mediated causes of death in the rising wolf population in Northern Italy, this study aims to: (i) investigate the prevalence of *Trichinella* spp. infection in these wolves; (ii) discuss if and how the return of this apex predator, after decades from extirpation, may modify the current low endemic status of *T. britovi* amongst the carnivore community in this renewed mountain ecosystem. In the background, there is a putative risk that the increased opportunities of infected carnivore scavenging by wild boars may result in a higher prevalence of *Trichinella* in this major bridge-host, and that the risk of wild boar-mediated transmission to humans may rise accordingly.

## 2. Materials and Methods

### 2.1. Study Area

The study was conducted in the western part of the Italian Alps, a mountain arch open to the east, encompassing the southern and western portions of two administrative regions, Piedmont and Aosta Valley. The study area covers 20,552 km^2^ and includes Turin, Cuneo and Aosta provinces. Elevations range from approximately 350 to 4800 m a.s.l, from the mesomediterranean to the nival elevation zone. Although impacted by global warming, climate may be still defined cold in winter and cool in summer, with mean temperatures on the valley floors of −5 °C to 4 °C in January and 15 °C to 24 °C in July. In winter, nearly all precipitation above 1500 m is in the form of snow, and the snow cover may last at 2000 m from mid-November to May [38].

In the study area, wolves share habitat with varied wild ungulate populations, including Alpine ibex (*Capra ibex*), Northern chamois (*Rupicapra rupicapra*), roe deer (*Capreolus capreolus*), red deer (*Cervus elaphus*), wild boar and the alien European mouflon (*Ovis aries musimon*). Livestock is also present, mostly in form of transhumant flocks and herds allowed to graze on alpine pastures from late May to October. Besides wolf, the community of mammalian carnivores in the study area is composed of red fox, by far the most abundant mesocarnivore, and by badger (*Meles meles*), stone marten (*Martes foina*), pine marten (*Martes martes*), stoat (*Mustela erminea*) and common weasel (*Mustela nivalis*) (http://www.sistemapiemonte.it/cms/privati/territorio/servizi/549-banche-dati-naturalistiche/2867-fauna/ accessed on 9 January 2023). As regards other canids, feral dogs are uncommon and the golden jackal (*C. aureus*) has been sporadically observed in recent years.

### 2.2. Wolf Sampling

The study was conducted from 2017 to 2022. Wolf carcasses were collected by wildlife conservation officers belonging to several partner institutions of EU-funded LIFE-WolfAlps Project (https://www.lifewolfalps.eu/ accessed on 9 January 2023), usually in response to an alert by citizens. All carcasses were stored at −20 °C and transported in plastic bags to the necropsy room of the Department of Veterinary Sciences, University of Torino, Italy, where they were processed. At post-mortem, the origin, sex, age, weight and the ultimate cause of death of each wolf were recorded on individual forms. Age of the animals was estimated from dentition and levels of wear on the teeth [39], and the wolves were classified into the following three classes: juveniles (<12 months), yearlings (12 to 24 months) and adults (>24 months).

### 2.3. Laboratory Analysis and Larval Biomass Estimation

Muscle samples were collected from diaphragm and stored at −20 °C until processed. The detection of *Trichinella* larvae was performed according to the artificial digestion protocol by Gamble et al. [40]. Specifically, samples of 10–40 g were processed in pools of three to five wolves, always observing the recommended ratio between the amount of sample and the volume of digestion liquid. If tested positive, an additional muscle sample of 5 g previously stored at −20 °C from each individual was digested separately, and the larvae per gram (LPG) determined under a stereoscopic microscope. Finally, larvae were washed several times in distilled water, recovered and stored in 96% ethanol. All positive samples were forwarded to the International *Trichinella* Reference Center at the Istituto Superiore di Sanitá, Rome (Italy), for identification at the species level by a multiplex PCR [4].

The *Trichinella* larval biomass of each positive wolf was estimated according to Badagliacca et al. [27]. In particular, the following parameters were retained from published data: (i) the body mass percentage of wolves corresponding to striated muscles, namely 0.460 in males and 0.435 in females; (ii) the LPG diaphragm coefficient (0.409). Accordingly, the formula to estimate the larval biomass of each positive wolf was:

[body mass × (0.460 or 0.435 according to sex)] × [LPG value × 0.409]

The total *Trichinella* larval biomass in the wolf population of the study area was calculated on the basis of: (i) *Trichinella* spp. prevalence and median intensity in this study; (ii) the mean body mass of a sample of 180 juvenile, yearling and adult wolves (20.6 ± 3.9, 25.5 ± 4.1, 30.8 ± 5 kg, respectively) necropsied at the Department of Veterinary Science, University of Turin [41]; (iii) the estimated number of wolves in Northwestern Italy (N = 680; CI95% = 602–44) derived from a recent wolf survey carried out in 2020–2021 [8]; (iv) the age structure (percentage of each age class) of a long-term monitored European wolf population, corresponding to 34% juveniles, 20% yearlings and 46% adults [42]. The underlying formula to estimate the total *Trichinella* larval biomass amongst wolves inhabiting the study area was:

[(estimated number of wolves in each age class × corresponding mean body mass) × (*Trichinella* prevalence)] × [median larval biomass in positive wolves])

Nominal data such as origin, sex and age were analysed by the Chi-squared test for possible associations with positive *Trichinella* infection in wolves, using a statistical significance level of ≤0.05.

## 3. Results

A total of 130 wolves of different age classes (adults, yearlings and juveniles) and sex (females and males) were considered (Table 2). Of these wolves, 60 originated from the province of Cuneo, 37 from Torino and 33 from Aosta.

**Figure 1:**
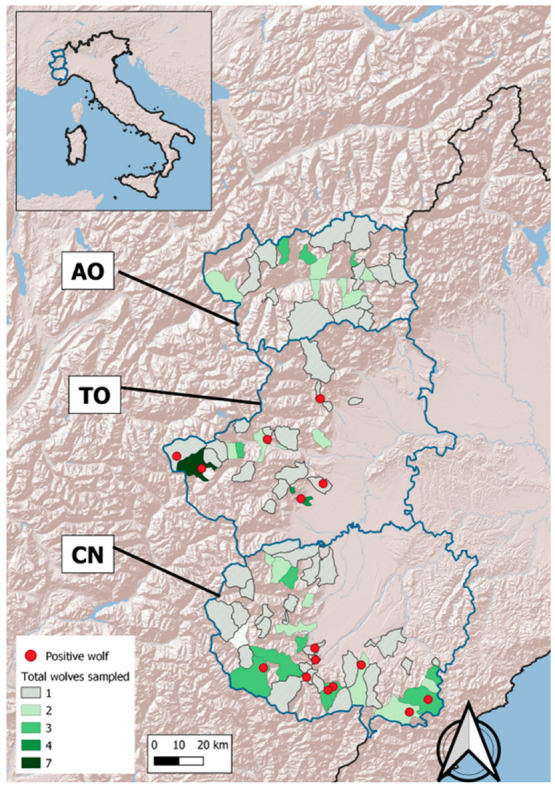
Map of the study area and its location with respect to Italy. Blue solid lines delimit the boundaries of the three provinces where the wolves tested were obtained: Aosta, Torino and Cuneo (from north to south). All *Trichinella*-positive individuals (red dots) were recovered in the Alps (zones in relief on the map).

*Trichinella* spp. larvae were detected in 15 out of 130 wolves (11.53%; 95%CI 7.12–18.17), originating from the Torino and Cuneo province, though not from the Aosta valley (Figure 1). Positive wolves are listed in Table 3.

Prevalence did not differ by sex or age class, whereas border line variation by wolf origin was found (*p* = 0.054). Nine isolates were identified as *T. britovi* by the International *Trichinella* Reference Center (Roma, Italy), whereas six were unfit for molecular typing due to defective sampling and preservation. The intensity of *Trichinella* infection varied between 0.8 and 45 LPG with a mean of 11.74 ± 11.16 LPG, and a median of 8.9 LPG (see Table 3 for individual raw data). Based on parameters and formulae made explicit in Material and Methods, (i) the estimated median number of muscle larvae in the carcass of *Trichinella* positive wolves was 36,116 LPG; (ii) wolves inhabiting the study area in 2020–2021 were supposed to harbor a total number of 2681.152 *Trichinella* larvae.

## 4. Discussion

Results of our survey, based on a large sample size, show that wolves recolonizing the Alps are exposed to *T. britovi* infection and may therefore contribute to the maintenance and spreading of this zoonotic parasite. This is in line with other similar studies in wolves in Italy [13,18,27], with the exception of a single case report of mixed *T. britovi* and *T. pseudospiralis* infection [37]. In other European countries, wolves were shown to harbor *T. britovi* [16,17,21,28,43] and, to a lesser extent, *T. nativa* [29,30,31,35], which is prevalent at high latitudes. As regards other taxa, *T. spiralis* has been unfrequently reported in Croatia [21], Finland [35], Central Balkans [14] and Germany [36].

To our knowledge, the *Trichinella* prevalence in this survey (11.53%) is amongst the lowest ones reported in wolves in Europe (see Table 1). In particular, most studies found a prevalence of over 30%, even much higher up to 60–97%, as in Western Russia [29,44], Estonia [30] and Latvia [45]. We cannot exclude that, to some extent, the prevalence we found may be underestimated, since only diaphragm samples were collected, and muscle samples have been stored at −20 °C in some cases for more than two weeks, with possible alteration of larvae sedimentation characteristics. While predilection sites of *Trichinella* have not been accurately determined in wolves, there is abundant literature on the preferable use of other muscles samples (e.g., the forelimb and the tibial muscles) for prevalence study of these parasites in foxes and other wild carnivores [46,47,48]. In previous similar surveys in Italy, prevalence ranged between 8.9 and 30.9%, with hotspots in central regions of the peninsula, where wolves never became extinct [13,27,37]. The low *Trichinella* prevalence in wolves in this study parallels the low prevalence amongst sympatric red foxes, the most abundant wild carnivore and the principal *Trichinella* maintenance host in Italy [49]. Of note, the prevalence of *Trichinella* spp. amongst foxes in our study area, which was as high as 40% in the late Fifties of the past century [19], has dramatically declined during the last decades of the past century [50] and is currently in the range of 0–3.5% [20,51,52]. Not surprisingly, *Trichinella* prevalence found in wolves in this study is higher, likely due to the apex predator rank and the longer life expectancy of wolves compared with foxes. Several studies of *Trichinella* spp. amongst wild carnivores in Europe also reported a higher *Trichinella* spp. prevalence in wolves and other large carnivores (e.g., the Eurasian lynx, *Lynx lynx*) compared to sympatric mesocarnivores [13,16,31,43]. An extreme example is the study conducted by Pozio et al. [29] in Western Russia, where *Trichinella* prevalence (mainly *T. nativa*) in wolves and sympatric foxes was 97.5 and 48.3%, respectively. In Central Italy, *T. britovi* prevalence was 27–31% in wolves and 4–5% in sympatric foxes [13,18]. As regards the Eurasian lynx, a study by Frey et al. [53] in the Swiss Alps showed that *T. britovi* prevalence was also remarkably higher than in sympatric foxes (27.3 and 1.6%, respectively).

Results of this and similar studies clearly point towards a sentinel role of the wolf, as sampling this top predator for *Trichinella* has proven to be an efficient way to estimate the risk that a zoonotic transmission may occur following the consumption of raw or undercooked wild boar originating from the same areas [13]. Interestingly, the only two outbreaks of human sylvatic trichinellosis recorded in Northwest Italy, both following the consumption of wild boar meat infected by *T. britovi* [54,55], occurred in alpine valleys in the provinces of Torino and Cuneo, where *Trichinella* positive wolves were also found in this study. Wolf centered surveillance of these zoonotic pathogens is nowadays facilitated by existing networks aiming to the conservation of this carnivore species in Europe, which generate a remarkable flow of wolf carcasses towards accredited veterinary institutions in view to monitor the natural and anthropogenic causes of death, and optimize the collection of biological samples for diverse purposes [56,57,58].

As for the epidemiological role played by the wolf in the *Trichinella* cycle in Italy and Europe, the actual significance of this apex predator in the maintenance of *T. britovi* is object of an open debate [13,21]. One opinion is that, in order to persist endemically, *T. britovi* (and any other sylvatic *Trichinella* taxon) requires an ecological chain composed of multiple interrelated carnivore hosts [59]. Under these circumstances, the enrichment of the carnivore community with an apex predator (e.g., the return of the wolf to the Alps) has the potential to generate new intraguild interactions [60,61,62,63] and new opportunities of cannibalism and interspecific scavenging and predation [64,65], eventually resulting in higher *Trichinella* prevalence and larval biomass, as occurred in Finland following colonization by another canid, the not native raccoon dog (*Nyctereutes procyonoides*) [35]. A second hypothesis is that foxes, the most investigated *Trichinella* host, would be capable to sustain the circulation of *T. britovi* independently of the presence of other infected carnivores, mainly through cannibalism, also referred as intraspecific scavenging [52]. An element supporting the fox centered hypothesis is the long-lasting *Trichinella* downward trend in foxes in Northern Italy, which has not been reversed since wolves made their comeback. On the other hand, similar as Badagliacca et al. [27], we register that the estimated *Trichinella* larval biomass harbored by wolves at approximately a quarter of century from their return, is in the range of approximately 2.5 million larvae, hence not negligible. Future similar surveys focused in wolves and sympatric foxes are warranted to monitor trends and possible shifts in the relative epidemiological significance of the one and the other host in the Alps. *Trichinella* larval biomass data in this study will serve as baseline for comparison.

While in eastern and northern Europe the wolf is unambiguously attributed a major role in maintaining the endemic status of *Trichinella* infection [14,16,17,21,22,28,29,44,45], the same does not necessarily apply in Southern Europe, where the return of this carnivore has apparently not reversed the long-term downward trend of *T. britovi* in the red fox, the most representative maintenance host of this taxon at the regional scale, as anticipated. The less obvious contribution that wolves recolonizing the Alps seem to offer to the maintenance and spreading of *T. britovi*, at least at the time being, may be attributed to one or a combination of several factors including: (i) the relatively short time since wolves re-established with reproductive packs in the study area; (ii) the low yearly number of infective wolf carcasses so far available to other scavengers, further reduced by the intentional removal of carcasses for monitoring purposes; (iii) possible reluctance of wolves towards cannibalism, a trophic behavior that has been poorly studied and, subsequently rarely documented and quantified [11], made apart some human-mediated exceptions, e.g., when wolf carcasses are repeatedly disposed of as baits to facilitate the killing of other wolves [44]. The last scenery is unlikely in the Alps since wolf is a protected species in the European Union and Switzerland; for this reason, wolf carcasses are made available to veterinary diagnostic centers or other scientific institutions, even when culling is allowed for mitigation of livestock depredation [66]; (iv) possible reluctance of other *Trichinella* maintenance hosts, in particular foxes, to feed on wolf carcasses, a neglected though epidemiologically intriguing issue. Previous experimental work on models other than wolf carcasses has shown that foxes make to avoid or delay the consumption of mesocarnivore carcasses [67,68], including conspecifics; (v) the so far limited mortality due to intra-specific aggressiveness, a density-dependent behavior which, in turn, could favor cannibalism. Expectedly, intra-specific aggressiveness will rise in the next future in parallel with the increasing alpine wolf population size, as occurred in North America under similar demographic conditions [69]; (vi) the influence of climate change, whose signal is stronger in mountain systems worldwide [70], implying a faster degradation of carcasses by necrophilic invertebrates [71] and, in winter, a shorter life expectancy of *Trichinella* larvae due to scarce snow precipitation and the consequent vanishing of the subnivium protective effect [72]. This, in turn, could narrow down the time window for effective transmission of *Trichinella* via the intraspecific and interspecific consumption of infected wolf carcasses.

Based on all the above, an address for future research could be to experimentally investigate the behavior of wolves towards the carcasses of conspecifics, foxes and eventually other carnivores (e.g., mustelids) reputed of lower epidemiological significance for maintenance of the sylvatic *T. britovi* cycle in Europe. In parallel, surveillance of *Trichinella* infection in the expanding wolf population in the Alps, and other areas under wolf recolonization, should be continued over time to define possible trends in prevalence and intensity, eventually suggesting a rising contribution of wolves to the maintenance and spreading of *T. britovi* in a context where the parasite had been able to maintain itself in foxes for several decades, or even centuries, after the human-mediated extirpation of any apex predators.

## 5. Conclusions

This is the first time that *T. britovi* is reported in wolves in the Italian Alps. Our results suggest that this apex predator, spectacularly expanding its population size since a few decades, deserves attention as a new possible maintenance host for *T. britovi* in the Alps. Data in this study will contribute to dynamically quantify the zoonotic risk in a changing epidemiological scenario characterized by a high diversity and abundance of wild carnivores and omnivores.

## Figures and Tables

**Table 1:** Prevalence and intensity studies of *Trichinella* spp. infection in the grey wolf (*Canis lupus*) in Europe. Surveys with limited sample size (≤15 wolves) and/or not reporting the identification of *Trichinella* muscle larvae to the species level were not included.

Country	Reference	Wolf Population (*)	Sample Size	Prevalence (%)	*Trichinella* spp.	LPG Mean (Range)	Period
Spain	[23]	NW Iberian peninsula	47	12.8	Tb	(0.3–5.75)	1996–1999
Italy	[24]	Italian peninsula	48	19.0	Tb	NA	1987–1993
Italy	[25]	Italian peninsula	25	28.0	Tb	NA	1991–1993
Italy	[18]	Italian peninsula	81	30.9	Tb	NA	1985–1995
Italy	[26]	Italian peninsula	67	8.9	Tb	NA	2008–2011
Italy	[13]	Italian peninsula	218	27.1	Tb	24.3 (0.2–250)	2004–2014
Italy	[27]	Italian peninsula	213	27.7	Tb	NA	2015–2020
Croatia	[21]	Dinaric–Balkan	67	31.0	Tb, Ts	7.6 (0.3–45.9)	1996–2007
Serbia	[16]	Dinaric–Balkan	116	46.5	Tb, Ts	11.4 (0.95–76)	2006–2013
Romania	[28]	Carpathian	35	31.0	Tb	11.3 (0.1–34)	2000–2005
Poland	[22]	Carpathian	21	54.5	Tb	3.75 (0.009–27)	1999–2015
Latvia	[17]	Baltic	23	100.0	Tb	3.2 (0.1–41.8)	2010–2014
Estonia	[29]	Baltic	24	75.0	Tn, Tb	NA	1992–1996
Estonia	[30]	Baltic	34	79.4	Tn, Tb	(0.01–44.9)	1992–1999
Finland	[31]	Karelian	18	33.0	Tn, Tb	2.9 (0.4–5)	1996–1998
Finland	[32]	Karelian	102	39.2	Tn, Tb, Ts	3.6 (0.18–57.5)	1999–2005
Finland	[32]	Karelian	85	34.1	Tn, Tb, Ts	NA	2011–2013
Sweden	[33]	Scandinavian	197	5.6	Tn, Tb	NA	2014–2019

(*) = according to Adamec et al. [34] Tb = *T. britovi*; Tn = *T. nativa*; Ts = *T. spiralis*. LPG = larvae per gram of muscle. NA = not available.

**Table 2:** Distribution of *Trichinella* spp. sampled wolves by sex and age classes. Juveniles are individuals <12 months, Yearlings between 12 and 24 months, Adults >24 months.

Sex	Adults (>24 Months)	Yearlings (12–24 Months)	Juveniles (<12 Months)	%
Males	33	12	22	51.53
Females	29	12	21	48.47
Total	62	25	43	

**Table 3:** Individual data referred to 15 wolves originating from Italian western Alps, that tested positive to *Trichinella* spp. in an artificial digestion test of diaphragm samples according to the European Commission Regulation (EC) no. 1375/2015 and 2022/1418.

Wolf Number	Year	Sex	Age Class	Body Mass (Kg)	Muncipality (Province)	Cause of Death	LPG	Total Estimated Number of Larvae
1	2017	F	J	21.1	Monastero di Lanzo (TO)	Intraguild killing	5.0	18,770
2	2017	M	Y	27	Frossasco (TO)	Road collision	0.9	4571
3	2018	M	A	31	Oulx (TO)	Train collision	8.9	51,907
4	2017	F	Y	25.2	Bardonecchia (TO)	Intraguild killing	10.7	49,973
5	2018	M	Y	26.2	Vernante (CN)	Train collision	7.0	34,505
6	2018	F	A	29.9	Roccasparvera (CN)	Road collision	11.5	61,176
7	2019	F	A	27.5	Piossasco (TO)	Illegal shot	5.5	26,909
8	2021	F	J	19	Vernante (CN)	Road collision	6.2	20,958
9	2021	M	A	35.6	Garessio (CN)	Road collision	13.7	91,759
10	2021	M	A	29.5	Ormea (CN)	Road collision	7.8	43,291
11	2021	F	J	11.4	Chiusa Pesio (CN)	Intraguild killing	26.8	54,356
12	2022	F	A	14	Chianocco (TO)	Starvation	14.5	36,116
13	2022	F	J	17	Vinadio (CN)	Road collision	11.8	35,689
14	2022	F	Y	25.5	Bernezzo (CN)	Road collision	0.8	3629
15	2022	F	J	17.5	Valdieri (CN)	Intraguild killing	45.0	140,108

F: female; M: male; J = juvenile; Y = yearling; A = adult. TO = Torino; CN = Cuneo.

## Data Availability

All relevant data are provided in the present study.
